# A method for calibrating measurement data of a micro air quality monitor based on MLR-BRT-ARIMA combined model

**DOI:** 10.1039/d3ra02408c

**Published:** 2023-06-12

**Authors:** Bing Liu, Peijun Jiang

**Affiliations:** a Public Foundational Courses Department, Nanjing Vocational University of Industry Technology Nanjing 210023 China Liub1@niit.edu.cn; b Automotive College, Sanmenxia Polytechnic Sanmenxia 472000 China

## Abstract

A micro air quality monitor can realize grid monitoring and real-time monitoring of air pollutants. Its development can effectively help human beings to control air pollution and improve air quality. However, affected by many factors, the measurement accuracy of micro air quality monitors needs to be improved. In this paper, a combined calibration model of Multiple Linear Regression, Boosted Regression Tree and AutoRegressive Integrated Moving Average model (MLR-BRT-ARIMA) is proposed to calibrate the measurement data of the micro air quality monitor. First, the very widely used and easily interpretable multiple linear regression model is used to find the linear relationship between various pollutant concentrations and the measurement data of the micro air quality monitor to obtain the fitted values of various pollutant concentrations. Second, we take the measurement data of the micro air quality monitor and the fitted value of the multiple regression model as the input, and use the boosted regression tree to find the nonlinear relationship between the concentrations of various pollutants and the input variables. Finally, the autoregressive integrated moving average model is used to extract the information hidden in the residual sequence, and finally the establishment of the MLR-BRT-ARIMA model is completed. Root mean square error, mean absolute error and relative mean absolute percent error are used to compare the calibration effect of the MLR-BRT-ARIMA model and other commonly used models such as multilayer perceptron neural network, support vector regression machine and nonlinear autoregressive models with exogenous input. The results show that no matter what kind of pollutant, the MLR-BRT-ARIMA combined model proposed in this paper has the best performance of the three indicators. Using this model to calibrate the measurement value of the micro air quality monitor can improve the accuracy by 82.4–95.4%.

## Abstract 2

The combined model proposed in this paper is superior to some commonly used air quality prediction models.

## Graphical abstract

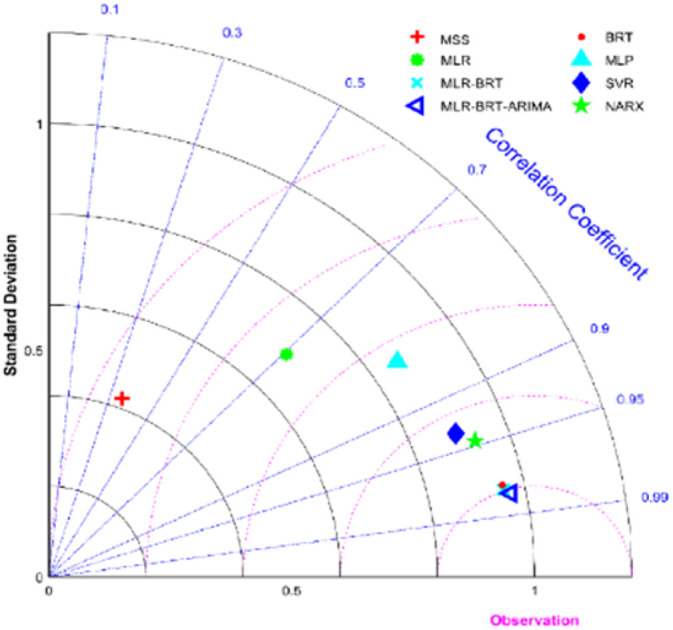

## 1. Introduction

With the rapid development of urbanization, urban air pollution has intensified, and the air pollution problem has become more and more harmful to human health. According to the World Health Organization, about 7 million people worldwide die from air pollution every year, and more than 90% of human beings breathe air pollutant concentrations higher than the limit set by the World Health Organization.^1,2^ In the city, the majority of pollution sources are man-made sources, which mainly include domestic pollution sources, industrial pollution sources, and traffic pollution sources. Long-term inhalation of polluted air by the human body can cause various diseases such as respiratory diseases and cardiovascular diseases. The harm of air pollution to human health has become one of the troubles affecting people's quality of life.^3,4^

Air quality monitoring stations are used by some developed cities to monitor air pollutants. These air quality monitoring stations are called reference sensor stations in this study. Although the pollutant concentration measured by the reference sensor station is relatively accurate,^5^ it is difficult to achieve grid monitoring in a certain area due to its high construction and maintenance costs. In addition, the measurement data of reference sensor stations also have the characteristics of lag in release, so it is difficult to realize real-time monitoring of pollutant concentrations. The emergence and development of micro air quality monitors effectively overcome these deficiencies of air quality monitoring stations. A micro air quality monitor is a commodity that can monitor outdoor air index conditions in real time. It samples the air according to the fluidity of the gas, the sampled gas reacts with the electrochemical sensor and generates an electrical signal corresponding to the gas concentration, and then the data monitoring result is obtained. Its production and maintenance costs are low, and it is easy to install and deploy. These advantages accelerate its grid deployment.^6,7^ The sites where the micro air quality monitors are deployed are called micro sensor stations in this study. The micro air quality monitor also has the advantage of easy reading, which makes it possible to monitor pollutants in real time. It can not only conveniently monitor the concentrations of PM_2.5_, PM_10_, CO, NO_2_, SO_2_, O_3_ (two aerosols and four gases) in the air, but also monitor meteorological parameters such as temperature, humidity, wind speed, air pressure, and precipitation. However, micro air quality monitors also have disadvantages such as short service life and poor linearity. In particular, the electrochemical sensor used in the micro air quality monitor will have a certain zero drift and span drift. In addition, changes in the concentration of unconventional gaseous pollutants (gas) and weather factors also have cross-interference on the sensor. These factors cause errors in the measurement data of the micro air quality monitor.^8^ The main objective of this study is to improve the measurement accuracy of micro sensor by establishing a statistical model to calibrate the data from micro sensor station near the reference sensor station using the measurement data from the reference sensor station. This will have positive implications for the development and popularization of micro air quality monitors.

Air quality forecasting has always been a research hotspot in academia. Scholars have carried out research on air quality from various aspects, including the discussion of factors affecting air quality and the prediction of the concentration of various pollutants. Table 1 is a summary of air quality forecasting model papers. Common air quality forecasting models are mainly divided into mechanism models and statistical models. The mechanism model is based on the scientific understanding of atmospheric physical and chemical processes, and uses meteorological principles to simulate the physical and chemical processes of pollutants, and uses the data generated by the simulation to predict the concentration of pollutants.^9–11^ Since the physical and chemical processes of the formation and propagation of pollutants are very complex, the computational complexity of the mechanism model is relatively high, and the accuracy of the model needs to be improved.

**Table 1:** A summary of air quality forecasting model papers

No.	Domains	Model	Duration	Ref.
1	Chemical domain	Chemometric model	2018	9
2	Chemical domain	Chemical transport model	2006–2007	10 and 11
3	Statistical domain	Multiple linear regression model	2001–2020	12, 13 and 20
4	Statistical domain	Time series model	2012–2019	14 and 15
5	Statistical domain	Hidden Markov model	2003–2013	16–18
6	Statistical domain	Gray prediction model	2020	19
7	Statistical domain	Artificial neural network	1999–2019	21–24
8	Statistical domain	Support vector machine	2015–2022	25–27 and 32
9	Statistical domain	Random forest	2012–2020	28–31

Statistical models establish air quality forecasting models mainly by analyzing characteristic factors related to changes in pollutant concentrations. Traditional statistical models include Multiple Linear Regression (MLR) models,^12,13^ time series models,^14,15^ hidden Markov models,^16–18^ gray prediction models,^19^ and so on. The multiple linear regression model has the advantages of simple structure, unique output results, and strong interpretability of the model. Based on the data from 2005 to 2016, multiple linear regression and geographically weighted regression models were used to assess the spatial distribution of PM_2.5_ in the eastern Indian state of Jharkhand over a ten-year period. Comparison of the results with the Akaike information criterion shows that the geographically weighted regression model performs better in predicting the spatial distribution of PM_2.5_.^20^ However, the factors affecting the concentration of air pollutants are very complex, and it is difficult for the multiple linear regression model to accurately reflect the nonlinear relationship between the concentration of air pollutants and various influencing factors. In recent years, with the improvement of computer computing power, artificial neural networks,^21–24^ support vector machines,^25–27^ random forests^28–30^ and other machine learning algorithms for air quality forecasting have gradually developed. Liu *et al.* used a combination of partial least squares and random forest methods based on data from air monitoring stations to achieve calibration of the measurement results of a micro air quality monitor. By comparing with some commonly used models, the combined model was found to be effective in improving the measurement accuracy of the micro air quality monitor measurements.^31^ Some researchers added geographical features such as population, land use, economy, pollution sources, and topographic parameters to the time series and established an air quality prediction framework for northern Taipei with the help of support vector machines, which has high accuracy in short-term time prediction of the region.^32^ Although the statistical model based on the machine learning algorithms cannot give the quantitative relationship between the input variable and the output variable, because it can simulate the nonlinear relationship between the input variable and the output variable and does not need to pre-set complex mathematical expressions, so machine learning algorithms tend to be more accurate than traditional statistical models.

**Fig. 1:**
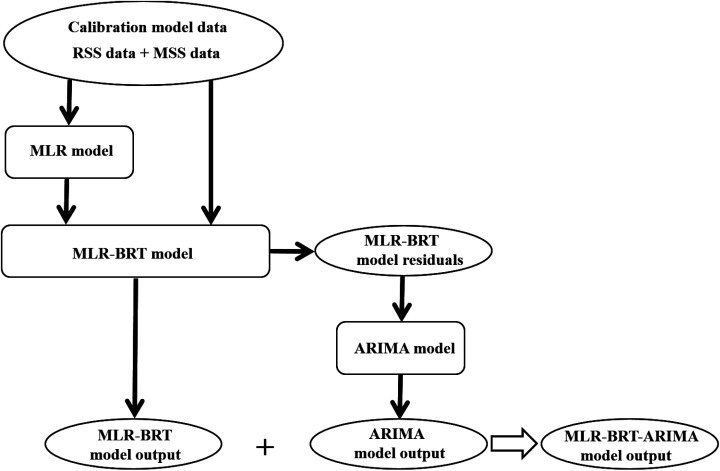
The flow chart of the regression process, where RSS represents the pollutant concentration measured at the reference sensor station and MSS represents the pollutant concentration measured at the micro sensor station.

Boosted Regression Tree (BRT) model is a data-driven random forest algorithm. It not only has a large tolerance for the data type, probability distribution and collinearity of predictors, but also can make comprehensive prediction of response variables on the basis of simulating the function characteristics of predictors. This study proposes a combined calibration model of multiple linear regression, boosted regression tree and AutoRegressive Integrated Moving Average (ARIMA) model, which we call the MLR-BRT-ARIMA model. This combined model combines the advantages of strong interpretability of MLR model and high accuracy of BRT model, and further extracts the information contained in the residuals by using ARIMA model, which can make MLR-BRT-ARIMA model with higher accuracy. Fig. 1 shows the modeling process of this study. Using this model, the measurement accuracy of pollutant concentrations can be improved, which provides a method reference for the calibration of the measurement data of the micro air quality monitor.

## 2. Data collection and analysis

### 2.1. Data source and preprocessing

At present, many large cities already have air quality monitoring stations, which can obtain monitoring data of air pollutants.^5^ In this study, two sets of air quality data from Nanjing were collected for statistical modelling (https://www.mcm.edu.cn/html_cn/node/b0ae8510b9ec0cc0deb2266d2de19ecb.html). The first set of data came from an air quality monitoring station in Jiangning District, Nanjing, which recorded the concentrations of six types of pollutants at this reference sensor station from November 14, 2018 to June 11, 2019. The first data set consisted of 4200 samples with a storage interval of 1 hour, and their measurements were considered as the reference values in this study. The second set of data comes from the micro air quality monitor used in this experiment, its location is juxtaposed with the reference sensor station, and the distance between the micro sensor station and the reference sensor station is no more than 10 meters. The second set of data measured by the micro air quality monitor had 234 717 samples with a storage interval of no more than 5 minutes for each sample. In addition, the second set of data provides not only the concentrations of six types of air pollutants, but also five meteorological parameters. Nanjing has a tropical monsoon climate with abundant rainfall, four distinct seasons, short spring and autumn, long winter and summer, and significant temperature differences between winter and summer. The area is a basin-like topography surrounded by mountains on three sides and water on one side, resulting in relatively poor atmospheric dispersion conditions. Under such natural conditions, various types of air pollution are associated with each other and interact with each other, which contributes to the composite pollution characteristics, continuous pollution characteristics and seasonal distribution characteristics of heavy air pollution in Nanjing.^33^

Before exploratory analysis, we first preprocess the data. Data that is less than 1/3 times the mean of the adjacent data before and after or more than 3 times the mean of the adjacent data before and after is identified as an outlier in this paper.^31^ For outliers and missing values, this paper deletes them. Then average the measurement data of the micro sensor station by hour to complete the correspondence with the data of the reference sensor station. Delete the data that cannot correspond to the micro sensor station and the reference sensor station. After preprocessing, a total of 4135 sets of corresponding data are obtained, which are shown in Table 2.

**Table 2:** Descriptive statistics of pollutant concentrations and meteorological parameters measured by reference sensor station and micro sensor station after pretreatment

Input variable	Ranges	Mean	Standard deviation	Skewness	Kurtosis	Coefficient of variation
PM_2.5_/μg m^−3^	1–216.9	64.1	37.3	0.988	0.701	0.582
PM_10_/μg m^−3^	2–443.3	102.4	65.3	1.476	2.862	0.637
CO/mg m^−3^	0.05–3.895	0.863	0.452	1.463	3.136	0.524
NO_2_/μg m^−3^	0.947–157.1	45.2	28.4	0.653	−0.259	0.628
SO_2_/μg m^−3^	1–651.3	19.4	18.7	12.781	342.11	0.965
O_3_/μg m^−3^	0.579–259	61.6	40.9	1.091	2.035	0.665
Wind speed/m s^−1^	0.133–2.387	0.7	0.346	0.862	0.748	0.494
Pressure/Pa	996.9–1039.8	1018.8	8.89	−0.093	−0.599	0.009
Precipitation/mm m^−2^	0–312.1	132.1	87	0.245	−0.728	0.659
Temperature/°C	−3.882–37.9	11.9	8.6	0.625	−0.399	0.724
Humidity/rh%	10.7–100	68.9	21.9	−0.487	−0.756	0.318

Among the six types of pollutants and five meteorological parameters, the standard deviation of precipitation is the largest at 87, and the standard deviation of wind speed is the smallest at 0.346. Since their means are quite different, the coefficient of variation can better reflect the degree of dispersion on the unit mean. The highest coefficient of variation of SO_2_ is 0.965, indicating that it has the highest average degree of dispersion, and the lowest coefficient of variation of pressure is 0.009, indicating that the average degree of dispersion of pressure is the lowest. Among the 11 variables, the coefficients of variation of pressure, humidity and wind speed are below 0.5, which indicates that their average dispersion is relatively low, while the other variables have a high average dispersion. Skewness is a measure of the direction and degree of skewness of a statistical data distribution. The skewnesses of pressure and precipitation are close to 0, and their distributions can be considered symmetric, while the skewnesses of O_3_, CO, PM_10_ and SO_2_ are all above 1, indicating that they have a severe right skewness. Kurtosis is a statistic that investigates the steepness or smoothness of the distribution of data. The kurtosis of O_3_, PM_10_, CO and SO_2_ all exceed 1, indicating that the distribution of their data is steeper than the normal distribution, and the absolute values of the kurtosis of the remaining variables are less than 1, indicating that the kurtosis of their distributions is close to the normal distribution.

### 2.2. Data exploratory analysis

Exploratory analysis is an in-depth and detailed descriptive statistical analysis of variables that facilitates further analysis of the data. The reference sensor station and micro sensor station data are averaged by day and a line graph is drawn to visually reflect the difference between the two.^20,34^

**Fig. 2:**
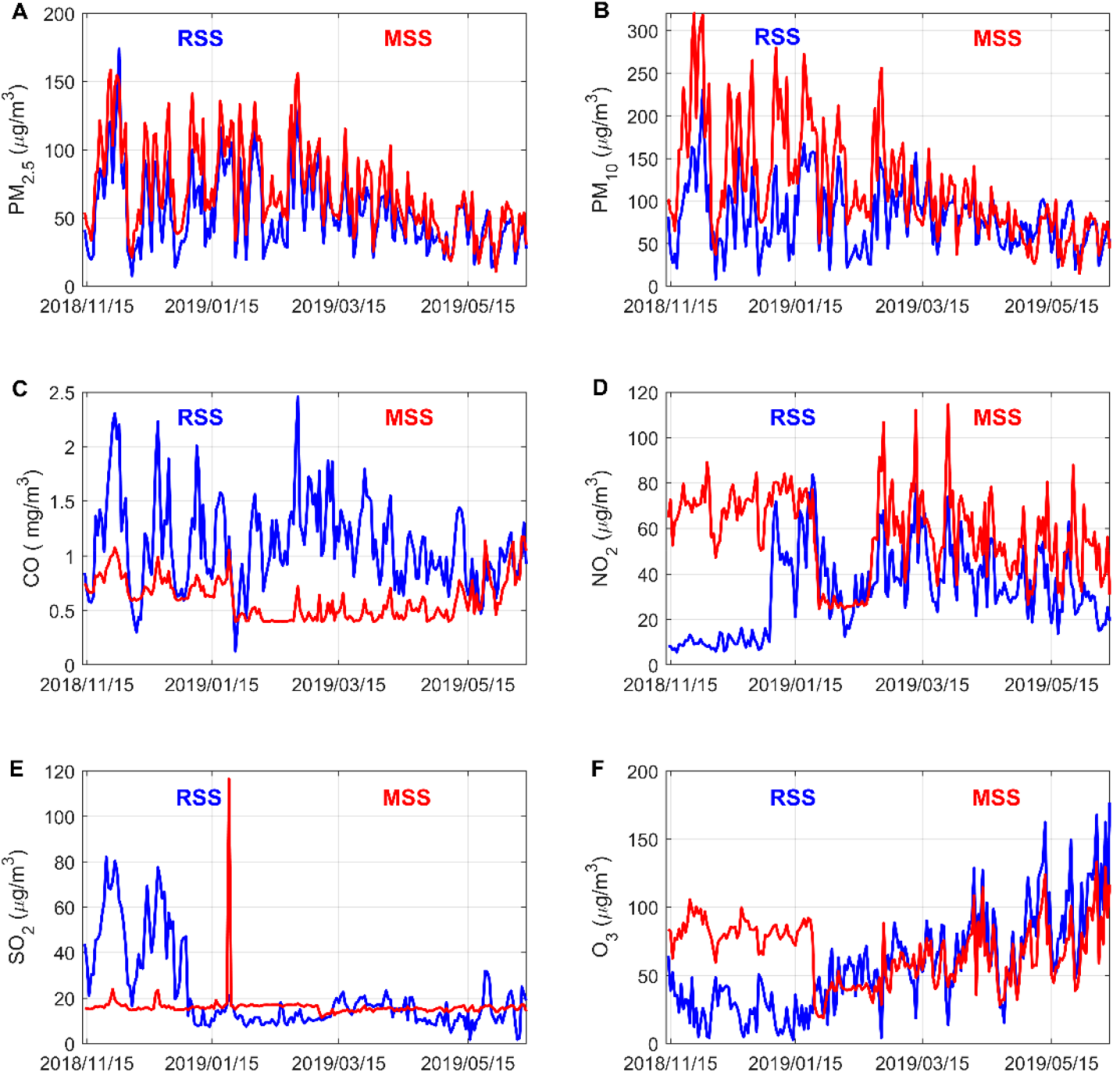
Comparison of daily average data of six types of pollutants at reference sensor station (RSS) and micro sensor station (MSS). Figures are generated using Matlab (version R2019a, https://www.mathworks.com/) [software].

It can be seen from Fig. 2 that the change trends of PM_2.5_ and PM_10_ concentrations measured by the reference sensor and micro sensor are basically the same, indicating that the micro air quality monitor has a high accuracy for the measurement of the concentrations of these two pollutants. The NO_2_ and O_3_ concentrations measured at the reference sensor and the micro sensor have large differences in the early stage and small differences in the later stage. The difference in CO and SO_2_ concentrations measured by the reference sensor and the micro sensor is large, indicating that the micro sensor has difficulty in accurately measuring the concentrations of these two pollutants. In general, the micro sensor differs in the accuracy of measurement of six types of pollutants.

**Fig. 3:**
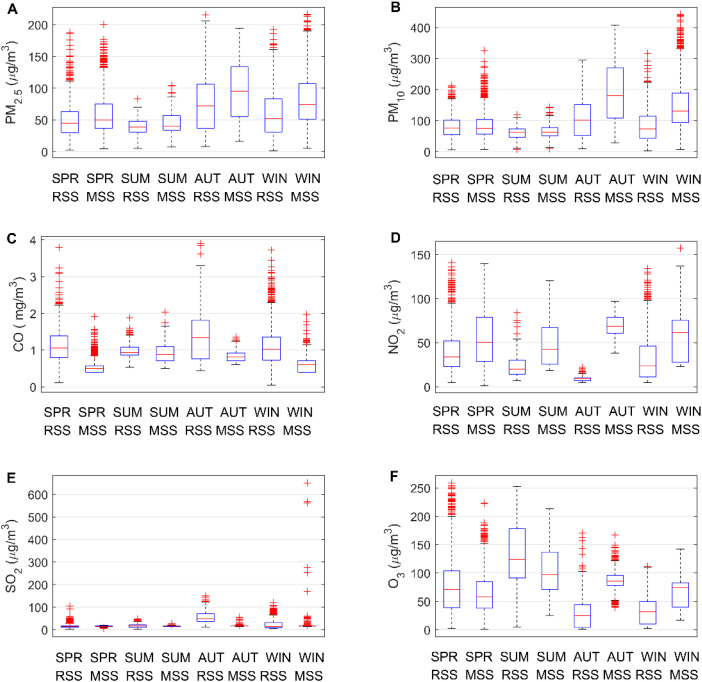
Comparing the concentration of six types of pollutants at reference sensor station (RSS) and micro sensor station (MSS) on a seasonal basis. Here SPR represents spring, SUM represents summer, AUT represents autumn, and WIN represents winter.

Fig. 3 is a boxplot of the six pollutant measurements categorized by season.^35,36^ The concentrations of PM_2.5_, PM_10_, CO, and SO_2_ pollutants are higher in autumn and winter. It is mainly due to lower precipitation in autumn and winter, resulting in slower diffusion of pollutants. In addition, affected by temperature, there are no air conditions conducive to the diffusion of pollutants in autumn and winter, which also leads to higher concentrations of these four pollutants in autumn and winter. The high NO_2_ concentration in spring may be related to lightning activity. Strong solar radiation and higher temperature in summer can easily cause photochemical smog and secondary ozone production, resulting in higher O_3_ concentration in summer. In addition, in different seasons, the climate parameters are different, and the measured values of the reference sensor and the micro sensor are significantly different, which also shows that the climate parameters will affect the measurement of the micro air quality monitor.^37^

The factors affecting the concentration of air pollutants are very complex, and each influencing factor also affects each other. The Pearson correlation coefficient is used to measure the correlation between two variables.^26,38^ In eqn (1), *x*_*i*_ and *y*_*i*_ respectively represent the *i*-th sample value of the two variables. The value range of the Pearson correlation coefficient is [−1,1]. When it is positive, it means that the two variables are positively correlated and when it is negative, it means that the two variables are negatively correlated. The degree of correlation between two variables increases with the absolute value of the Pearson correlation coefficient.

**Fig. 4:**
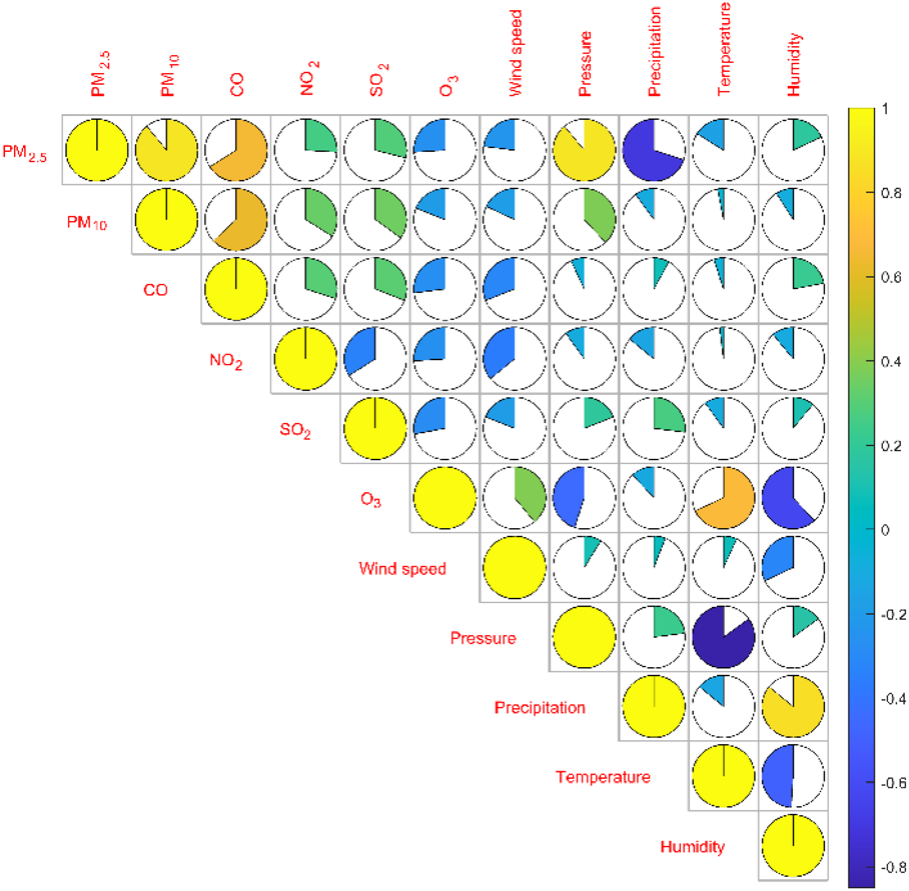
Pearson correlation coefficient matrix color block diagram between the concentration of two aerosols and four gases and climate factors.

It can be seen from Table 3 that the correlation coefficient between PM_2.5_ and PM_10_ is 0.89, which is the highest degree of positive correlation, indicating that their concentration trends are highly consistent. The correlation coefficient between temperature and air pressure is −0.85, which is the highest degree of negative correlation, indicating that air pressure decreases as temperature increases. The matrix color block diagram can intuitively show the correlation coefficient between the variables. In Fig. 4, the area of the sector represents the absolute value of the correlation coefficient, light color represents positive correlation, dark color represents negative correlation, and the lighter the color, the larger the correlation coefficient.1
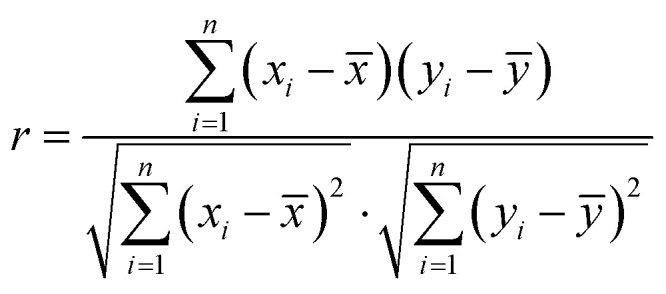


**Table 3:** Pearson linear correlation coefficient between the concentrations of six types of air pollutants measured at reference sensor station and five meteorological parameters measured at micro sensor station (band * indicates significant correlation at a significant level of 0.05)

Variable	PM_2.5_	PM_10_	CO	NO_2_	SO_2_	O_3_	Wind speed	Pressure	Precipitation	Temperature	Humidity
PM_2.5_	1.00	0.89*	0.66*	0.26*	0.29*	−0.26*	−0.23*	0.89*	−0.70*	−0.16*	0.18*
PM_10_		1.00	0.63*	0.34*	0.35*	−0.19*	−0.18*	0.38*	−0.10*	−0.03*	−0.09*
CO			1.00	0.30*	0.31*	−0.27*	−0.31*	−0.07*	0.08*	−0.05*	0.22*
NO_2_				1.00	−0.34*	−0.26*	−0.36*	−0.10*	−0.14*	−0.02	−0.11*
SO_2_					1.00	−0.28*	−0.19*	0.19*	0.27*	−0.10*	0.11*
O_3_						1.00	0.39*	−0.45*	−0.12*	0.68*	−0.62*
Wind speed							1.00	0.09*	0.06*	0.07*	−0.32*
Pressure								1.00	0.23*	−0.85*	0.15*
Precipitation									1.00	−0.14*	0.86*
Temperature										1.00	−0.49*
Humidity											1.00

## 3. Establishment of sensor calibration model

### 3.1. Description of statistical models

The idea of boosting method originally came from the “AdaBoost.M1” classification algorithm proposed by Freund and Schapire in 1997.^39^ It is mainly used to improve the performance of classification trees in binary classification problems. In 2000, Friedman extended the idea of boosting method to regression problem and combined it with the method of regression tree, and proposed the algorithm of boosted regression tree.^40^ The basic idea of BRT is to repeatedly apply the regression tree algorithm to the continuously adjusted training data to obtain a set of regression trees, and then perform a weighted average of the set of regression trees to obtain a final regression tree. Both theoretical research and practical application show that BRT can improve the prediction accuracy of regression tree.

Before understanding the BRT algorithm, let's review the basic concepts of regression trees. Regression tree is one of the most widely used algorithms in data mining and machine learning. When fitting the data, it first divides the joint space of the predictor *X* into non-overlapping *J* small regions *R*_*j*_, which are called the terminal nodes (or leaves) of the tree, and then fit a constant *γ*_*j*_ to each small region as the predicted value of the response variable *y* in this small region (eqn (2)). For a definite division *R*_1_, *R*_2_, …, *R*_*J*_, the regression tree model can be expressed as eqn (3). At this point, *L*(·) as a loss function can be used to represent the measurement error of the regression tree for the training data. In regression trees, the most commonly used loss function is the squared loss function *L*(*y*, *f*(*x*)) = (*y* − *f*(*x*))^2^. The two sets of basic parameters of the regression tree are the small area *R*_*j*_ and the corresponding constant *γ*_*j*_ on the small area, which are unified as *Θ*. Eqn (4) is the criterion for the estimation of parameter *Θ*, where *L*(·) is the loss function. This generates a regression tree (eqn (5)), where the parameters of the regression tree are the ones that minimize the sum of the residuals of the training samples. In this paper the residuals refer to the difference between the actual observed values and the model fitted values.

2 *x* ∈ *R*_*j*_ ⇒ *f*(*x*) = *γ*_*j*_

3
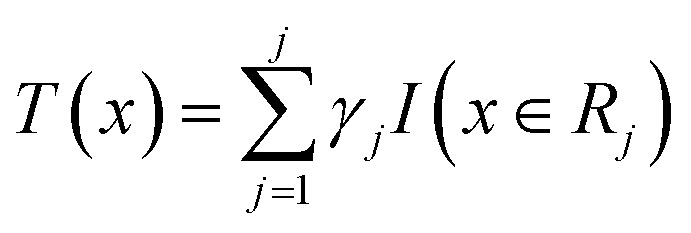
4
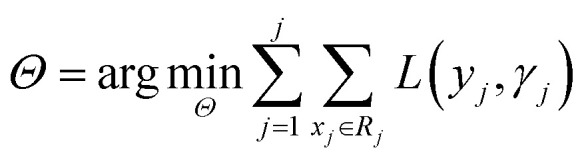
5
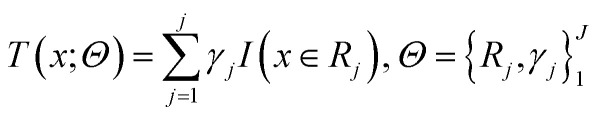


Compared with several other popular data mining algorithms, regression tree has the advantages of fast calculation, strong interpretability (if the number of leaves *J* is relatively small), and invariance to monotonic transformation of predictors. At the same time, the tree is not sensitive to outliers, and the tree can automatically select variables during the generation process. Due to the above advantages, the tree can be called an “off the shelf” method, which can be used directly for data processing without the need for time-consuming data preprocessing. But a major disadvantage of regression trees is that the predictions are not accurate enough. We know that the mean squared error can be decomposed into: MSE = Var + Bias under the squared error loss function. The inaccurate prediction of a regression tree is mainly because of its large variance, not because of bias. The boosting method significantly reduces the variance of the regression tree by performing a weighted average on the regression tree, thereby greatly improving the prediction accuracy of the tree.

BRT is a combination of *M* regression trees through an additive model, and eqn (6) is its general form. Eqn (7) is the parameter estimation criterion for each tree, where *L*(*y*_*i*_, *f*_*m*−1_(*x*_*i*_) + *T*(*x*_*i*_, *Θ*_*m*_)) = [(*y*_*i*_ − *f*_*m*−1_(*x*_*i*_)) − *T*(*x*_*i*_, *Θ*_*m*_)]^2^. At this point, *T*(*x*, *Θ̂*_*m*_) is the regression tree with the best fitting effect on the residual of the previous step under the squared loss.^41,42^6
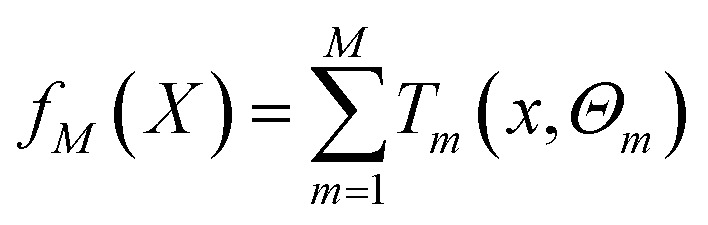
7
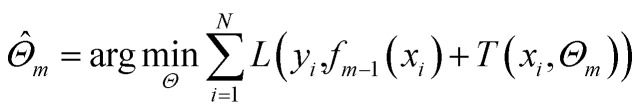


ARIMA model is a time series combination model that combines autoregressive process and moving average process, generally written as ARIMA (*p*, *d*, *q*), where *p* is the lag order of the autoregressive process, *d* is the order of making the time series stationary difference, and *q* is the lag order of the moving average process. The stationary series after differencing the series, we can use the ARIMA model to fit the prediction. Eqn (8) is the mathematical description of the ARIMA model, where *y*_*t*_ is the original time series, and Δ^*d*^*y*_*t*_ represents the stationary series of *y*_*t*_ after *d* differences. *θ*_0_ is a constant, *ϕ*_*i*_ is the coefficient of the autoregressive lag term Δ^*d*^*y*_*t*−1_, Δ^*d*^*y*_*t*−2_, …, Δ^*d*^*y*_*t*−*p*_, *ε*_*t*_ represents the error term, and the error sequence is assumed to be a Gaussian white noise sequence with zero mean and variance *σ*^2^. *θ*_*i*_ is the coefficient of the moving average lag term *ε*_*t*−1_, *ε*_*t*−2_, …, *ε*_*t*−*p*_.^43,44^8
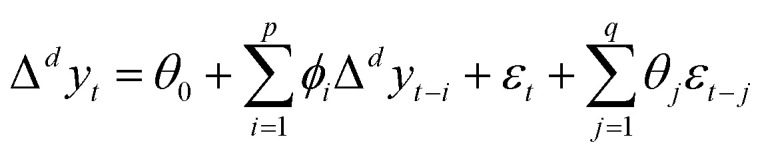


### 3.2. MLR calibration model

The multiple linear regression model is a classic statistical model that is often used to predict pollutant concentrations.^12^ The key to building a multiple regression model is the choice of independent variables. Too few variables are selected into the model, and the effect of the regression equation is definitely not good. If more variables are introduced into the model, variables that are not important to the dependent variable may be introduced into the model. Some variables have large overlap with other variables, which can also lead to poor model stability and affect the use of the model. The forward method, backward method, and stepwise regression are all more commonly used methods for variable selection.^34^ The backward method first establishes a full-variable model, then gradually eliminates the independent variables that are not statistically significant, and finally completes the construction of the regression model. It has the advantage that the selection of independent variables is more comprehensive and can effectively avoid the omission of effective variables. In order to introduce more variables into the air pollutant calibration model, the backward method was used in this study to select the independent variables.

Before using the backward method to build the multiple regression model, the 4135 samples were divided into training and test sets in a ratio of approximately 3 : 1. A total of 3100 samples are included in the training set to build the multiple regression model, and 1035 samples are included in the test set to test the calibration effect of the calibration model. The construction process of the six types of air pollutant concentration calibration models is similar. This paper randomly selects CO as an example to describe the calibration model construction process, and the other pollutant concentration calibration models can be obtained similarly. We take the CO concentration measured at the reference sensor station as the dependent variable, the two aerosols and four gas concentrations and five meteorological parameters measured at the micro sensor station as the independent variables, and use the backward method to select variables. With the help of linear regression routines from SPSS20.0, the remaining 10 variables of the 11 variables measured by the micro sensor station were introduced into the multiple regression model of CO concentration except for the SO_2_ concentration. In the significance test of the regression coefficient, the 10 variables introduced into the model all had a significant impact on the CO concentration at the significant level *α* = 0.05. The *F* value of the regression coefficients were 32.8, corresponding to a *P* value of 0.00, indicating that the independent variables introduced into the model had a significant impact on the CO concentration as a whole. The coefficient of determination *R*^2^ of the model was 0.515, indicating that 51.5% of the variation in CO concentration could be explained by the variation in the independent variables. Table 4 shows the multiple linear regression models of the concentrations of six types of air pollutants.

**Table 4:** Multiple linear regression model of six types of air pollutant concentrations. In the model, the dependent variable is the concentration of the six pollutants at the reference sensor station, and the independent variables are the observations of the micro sensor station (“—” represents the variables eliminated in the model)

Independent variable	PM_2.5_	PM_10_	CO (×10^−2^)	NO_2_	SO_2_	O_3_
Constant	436.4	1231.9	2539.8	1223.7	−345.4	−722.3
PM_2.5_	0.784	0.755	0.835	0.556	−0.168	0.951
PM_10_	−0.343	0.118	−0.08	−0.271	0.129	−0.566
CO	−0.412	28.7	41.4	—	32.2	−15.7
NO_2_	8.64	0.353	0.221	0.426	0.051	−0.603
SO_2_	—	0.085	—	—	−0.057	0.073
O_3_	—	0.032	0.096	−0.098	0.099	0.561
Wind speed	−0.031	—	−12.8	−17.6	−5.57	15
Pressure	0.076	−1.14	−2.43	−1.12	0.331	0.741
Precipitation	−0.182	−0.08	0.035	−0.031	0.018	0.01
Temperature	0.032	−1.16	−2.07	−1.6	—	2.63
Humidity	−1.3	−1.11	−0.335	−0.639	—	−0.223
*F* value	3290	1333.4	32838.6	391.3	239.3	1142.1
*R* ^2^	0.906	0.812	51.5	0.533	0.411	0.803

### 3.3. MLR-BRT combined calibration model

The multiple linear regression model can quantitatively analyze the linear relationship between pollutant concentrations and various influencing factors, but it cannot accurately reflect the nonlinear relationship between them. The BRT model significantly reduces the variance of the regression tree by performing a weighted average of the regression trees, thereby greatly improving the calibration effect. It is used in this study to find the nonlinear relationship between air pollutant concentrations and various influencing factors.

The regression learning toolbox that comes with Matlab2019 is used in this paper to build the boosted regression tree model. The dependent variable in the boosted regression tree model is the measured values of air pollutants at the reference sensor station grouped according to the previous section, and the independent variable is the measured value of the micro sensor station and the fitted value of the multiple regression model. This multivariate regression and boosted regression tree combination model is referred to herein as the MLR-BRT combination model. In the boosted regression tree model there are three main parameters, which are minimum leaf size, number of learners and learning rate. Minimum leaf size is a parameter that specifies the minimum number of training samples used to calculate the response of each leaf node. It will not achieve high training accuracy if it is too small, and it will tend to overfit if it is too large. Many learners can produce high accuracy, but fitting can be time-consuming. The learning efficiency determines the training time required for the model to reach the optimal level. If the learning efficiency is too small, the convergence speed will be slow, and the training time will be longer; if the learning efficiency is too large, noise is likely to be generated during sampling, resulting in reduced function smoothness and poor stability.

**Fig. 5:**
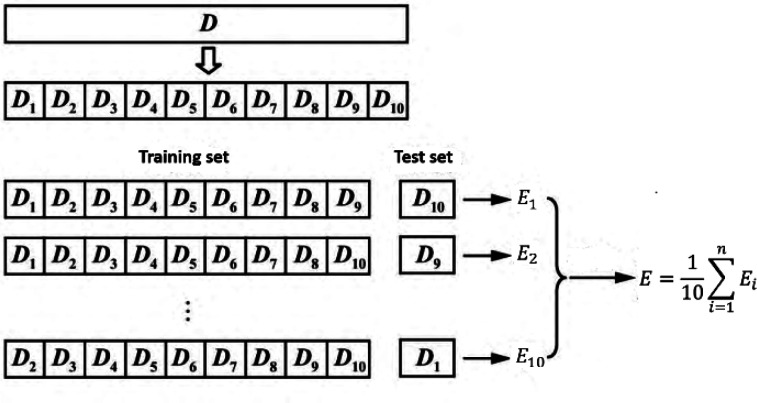
10-fold cross-validation process description and implementation.

Grid search and K-fold cross-validation were used to select the three parameters of CO's MLR-BRT model. The optimization range of minimum leaf size is 1–19, and the step size is 2; the optimization range of number of learners is 300–800, and the step size is 50; the optimization range of learning rate is 0.02–0.2, and the step size is 0.02. The mean deviation of the K-fold cross-validation was used to determine the final parameter values. K-fold cross-validation means that the data set is randomly divided into K parts, and K-1 parts are selected as the training set each time, and the remaining 1 part is used as the test set. After obtaining K models, the average test effect of these K models is used as the final model effect. In this paper, *k* = 10 is selected, and Fig. 5 is the structure diagram of *k*-fold cross-validation. Based on 10-fold cross-validation, the minimum leaf size is set to 13, the number of learners is set to 650, and the learning rate is set to 0.18.

**Fig. 6:**
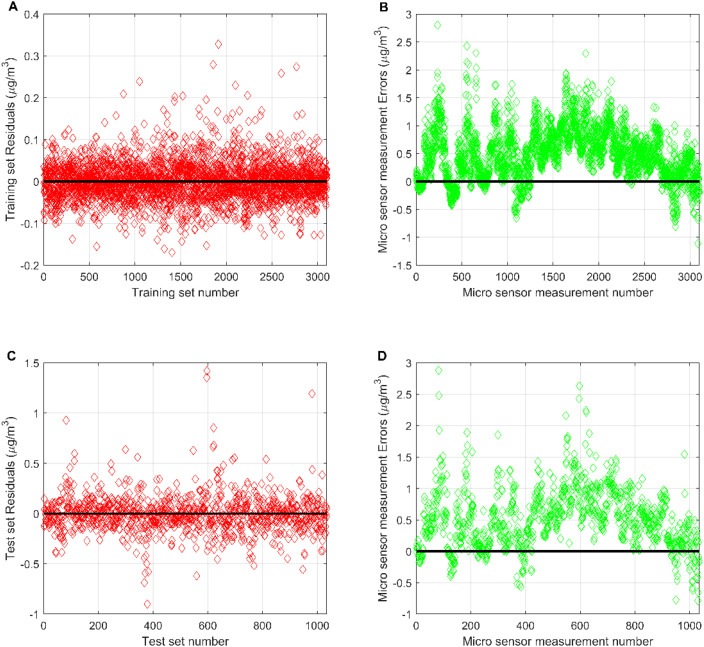
(A) Residuals of the MLR-BRT calibration model on the training set; (B) the measurement error of the micro sensor at the number corresponding to the training set of the MLR-BRT calibration model; (C) residuals of the MLR-BRT calibration model on the test set; (D) the measurement error of the micro sensor at the number corresponding to the test set of the MLR-BRT calibration model.

Fig. 6 compares the micro sensor station measurements of CO with the output from the MLR-BRT model. The CO measurement errors of micro sensor stations are concentrated in [−1, 2], and the number of sample points with positive errors is obviously more than the number of sample points with negative errors, indicating that the CO concentrations measured by the micro sensor station are lower than the CO concentrations measured by the reference sensor station. By comparing the mean values of both, it can be found that the mean value of CO concentration measured by the micro sensor station is 0.502 mg m^−3^ lower than the mean value of CO concentration measured by the reference sensor station. The training set error of the MLR-BRT model is concentrated at [−0.2, 0.2], and the test set error is concentrated at [−0.5, 0.5]. The errors on both the training and test sets are uniformly distributed around zero. This calibration model has obvious improvements to the CO concentration measurements at micro sensor station.

### 3.4. MLR-BRT-ARIMA combined calibration model

Although the MLR-BRT model has improved the measurement accuracy of micro sensor stations, the time factor is not added to the model in the modeling process. Since the electrochemical sensor will have zero drift and span drift over time, it is necessary to mine the time-related information hidden by model errors. Commonly used residual information extraction and correction methods include local analog approximation, vector error correction, periodic extrapolation, Bayesian vector method, and ARIMA model.^21^ Compared with other methods, ARIMA model not only can describe the random time series data well and eliminate the errors caused by the drift of micro sensor over time, but also has the advantages of simple and efficient structure.

The key to the ARIMA model is the stationarity of time series data. The stationarity of a time series refers to the fact that the statistical characteristics of the time series do not change over time. It can be seen from Fig. 6 that the residual of the MLR-BRT model of CO is a sequence with basically no trend. The observations in the sequence generally fluctuate at a fixed level, and it can be considered a stationary sequence. Therefore, the number of differences takes *d* = 0. In the ARIMA (*p*, *d*, *q*) model, *p* and *q* can be determined by Akaike Information Criterion and Bayesian Information Criterions. With the help of time series forecasting routines from SPSS20.0, the order *p* = 1, *q* = 1 of the ARIMA model of the CO residual was determined, and the modified model of the CO residual time series data was ARIMA (1, 0, 1). Finally, a white noise test for the ARIMA model of CO is also required. The Ljung–Box test is used in this paper to test whether the autocorrelation of the residual series of the ARIMA model is significant, that is, whether the residual series of the ARIMA model is white noise. Its original hypothesis is that each value of the residual series is independent. The test results show that the Ljung–Box Q statistic is 16.51, the corresponding *p* value is 0.418, and the residual data of this model is white noise data.^26,45,46^ The final CO calibrated value is obtained by adding the fitted value of the ARIMA model and the fitted value of the MLR-BRT model. At this point, the MLR-BRT-ARIMA combined calibration model of CO has been established, and the MLR-BRT-ARIMA combined calibration models of other pollutants can be given similarly.

**Fig. 7:**
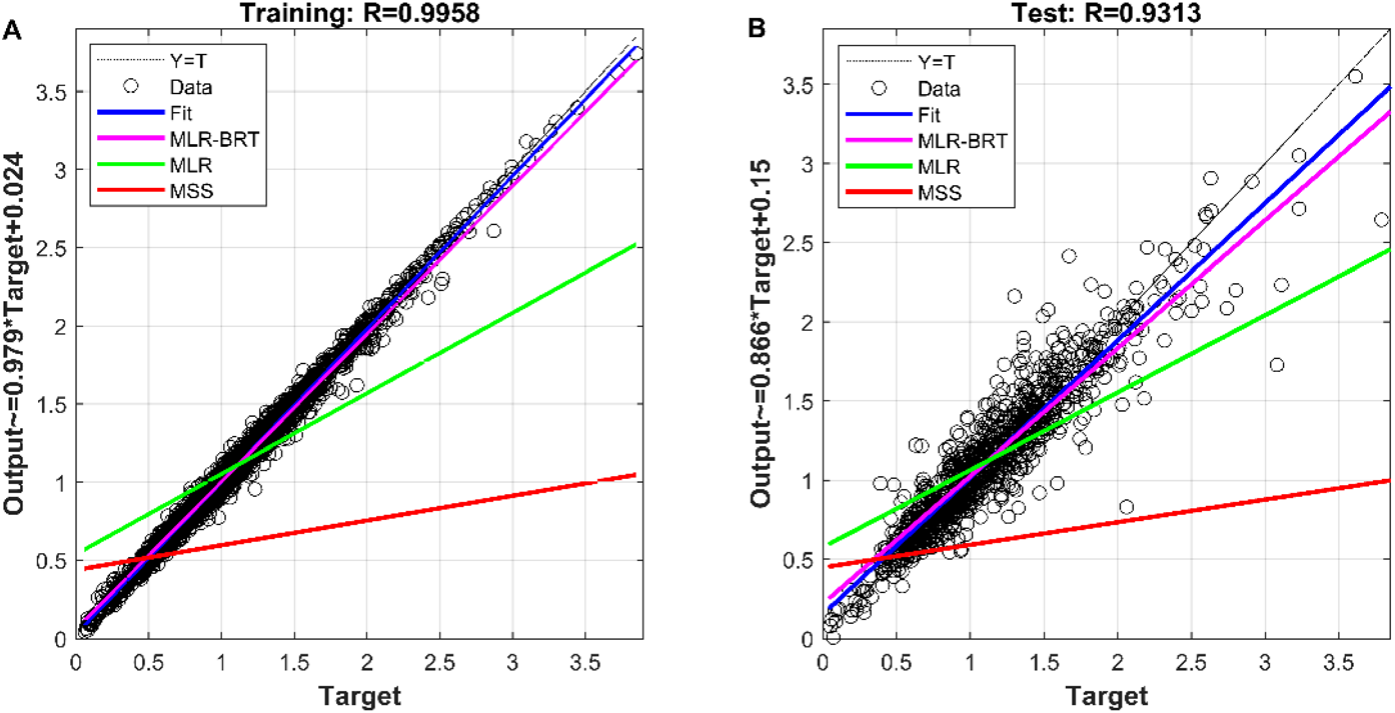
(A) The fitting effect of CO's MLR-BRT-ARIMA model on the training set; (B) the calibration effect of CO's MLR-BRT-ARIMA model on the test set.

**Fig. 8:**
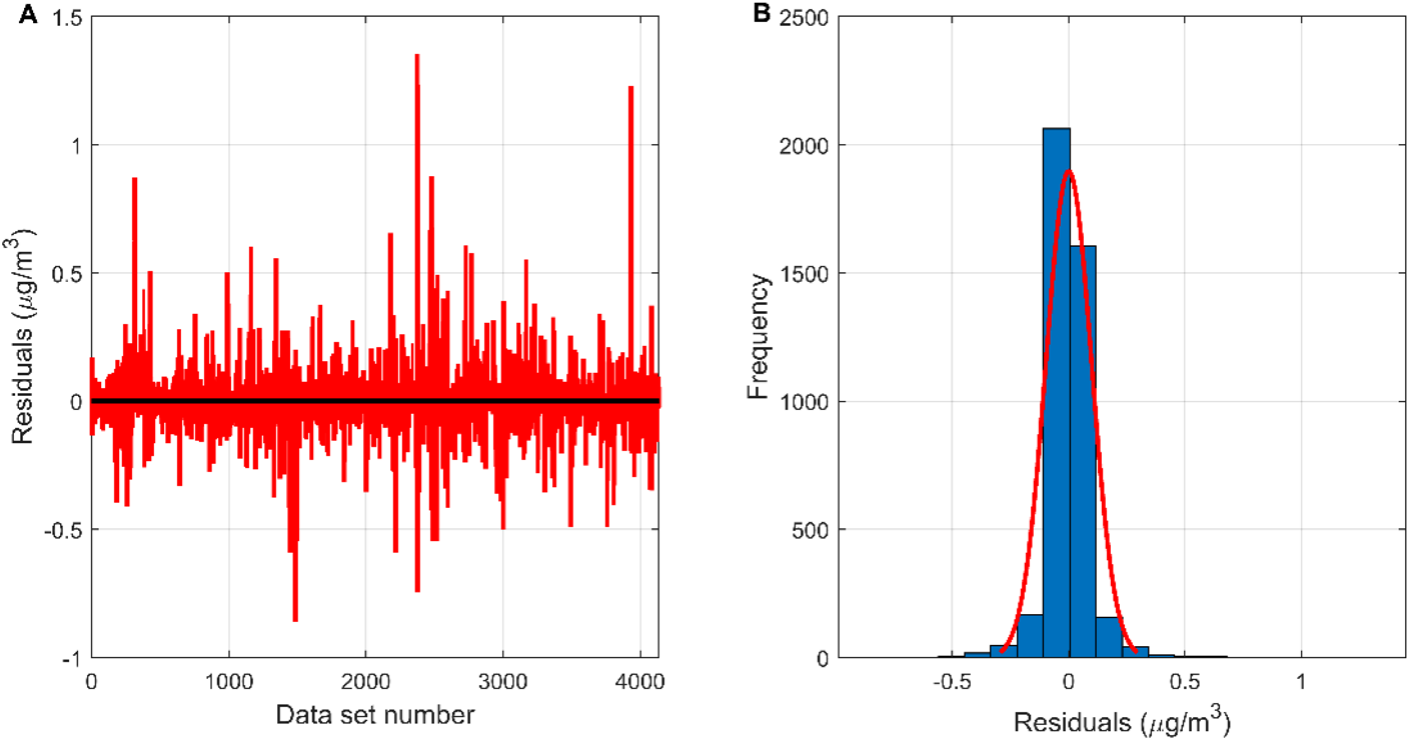
(A) The residual plot of the MLR-BRT-ARIMA model; (B) the residual histogram of the MLR-BRT-ARIMA model.

The measured value of the reference sensor is the target of the measured value of the micro sensor and the output value of each model. It is viewed as the independent variable, and the measured values of the micro sensor and the output values of each model are used as the dependent variables to build the regression model, and the regression effect is shown in Fig. 7. The correlation coefficients between the MLR-BRT-ARIMA model output values and the target values exceeded 0.93 for both the training and test sets, and the coefficients of both regression models were close to 1, indicating a strong correlation between the MLR-BRT-ARIMA model output values and the reference sensor measurements. In addition, the regression lines of the training set and the test set are greatly improved compared with the regression lines of the micro sensor station, indicating that the calibration model has a good effect on the micro sensor data quality. Residual testing is also an important step in statistical modeling. It can be seen from Fig. 8 that there are 3575 residuals of the model in [−0.1, 0.1], accounting for 86.5%, and 4111 residuals in [−0.5, 0.5], accounting for 99.4%. In the test set, there are 580 residuals in [−0.1, 0.1], accounting for 56.0%, and 1011 residuals in [−0.5, 0.5], accounting for 97.7%. The residual items are randomly and uniformly distributed around the 0 point, and the overall distribution is normal.

## 4. Discussion

In order to determine whether the trained model has good performance, the output of the model on the test set needs to be evaluated. The MLR-BRT-ARIMA combined model given in this study performed a good calibration for the CO measurement concentration of the micro air quality monitor. In addition, MultiLayer Perceptron neural network (MLP), Support Vector Regression machine (SVR) and Nonlinear AutoRegressive models with eXogenous inputs (NARX) are also frequently used to calibrate CO measurement concentration of micro air quality monitor.^22,26,47^ The Taylor diagram is used in this paper to visually compare the calibration effects of each calibration model.

Taylor diagram was first proposed by Karl E. Taylor in 2001 and is a visual polar diagram. It can simultaneously integrate standard deviation, centered root mean square difference and correlation coefficient on a polar plot. In the Taylor diagram, the scatter points represent different models, the horizontal and vertical axes represent the standard deviation, the dashed line represents the centered root mean square difference, and the radial line represents the correlation coefficient. Eqn (9) and (10) are expressions for standard deviation and entered root mean square difference, where *w*_*i*_ is the model fitted value, *w̄* is the mean of *w*, *y*_*i*_ is the reference value, and *ȳ* is the mean of *y*.9
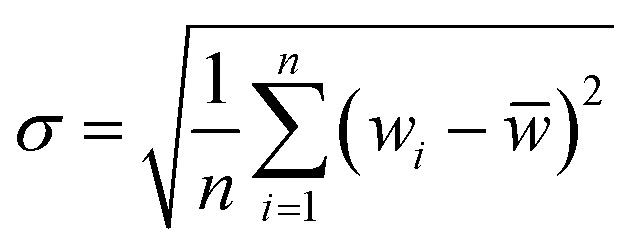
10
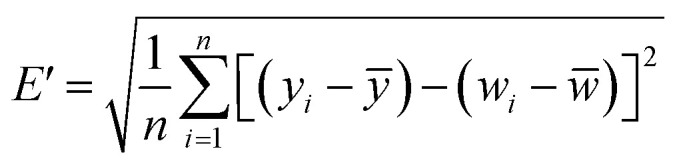


**Fig. 9:**
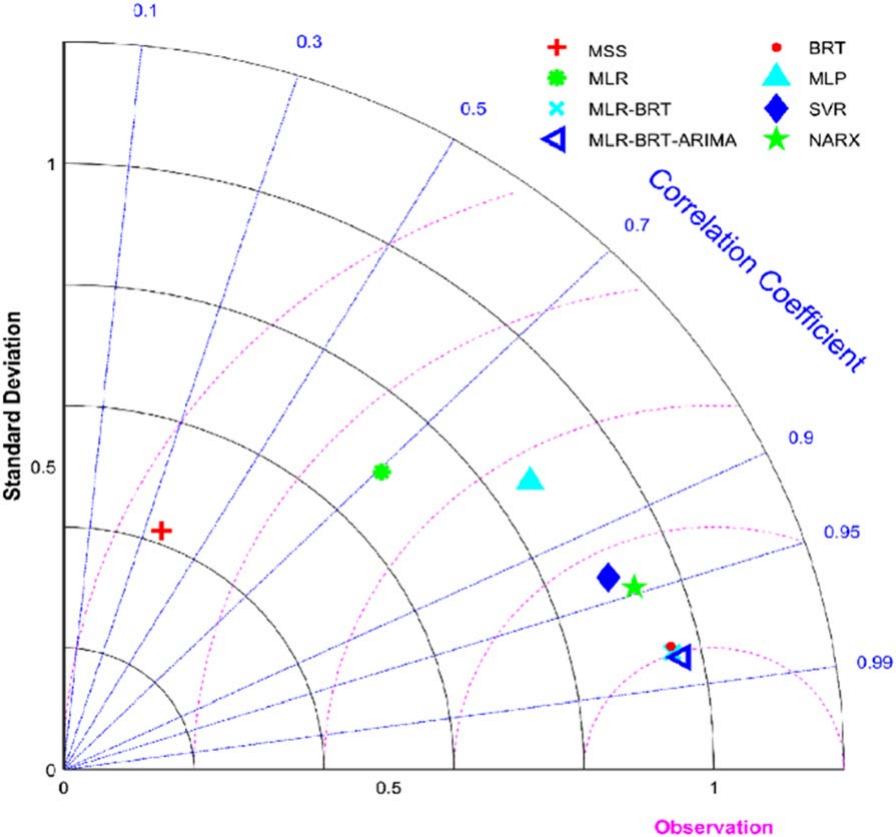
Taylor diagrams of the calibrated values CO concentration for the seven calibration models and the measured value of the micro sensor station, where MSS represents the micro sensor station.

It can be seen from Fig. 9 that the MLR, MLP, SVR and NARX models can calibrate the CO concentration of the micro sensor station, but the calibration effect needs to be improved. The BRT, MLR-BRT and MLR-BRT-ARIMA models have better calibration effects on the CO concentration measurement accuracy of the micro sensor station. In terms of the Pearson correlation coefficient, the correlation coefficient between the micro sensor station measurements and the reference sensor station measurements is 0.36, which is a low correlation, while the correlation coefficient between the fitted values of MLR-BRT-ARIMA model and the reference sensor station measurements is 0.98, which is a high correlation. In terms of standard deviation, the ratio of the standard deviation of the micro sensor station measurements to the standard deviation of the reference sensor station measurements is 0.429, while the ratio of the standard deviation of the fitted values of the MLR-BRT-ARIMA model to the standard deviation of the reference sensor station measurements is 0.97. It can be seen intuitively that the MLR-BRT-ARIMA combined model given in this paper has the best calibration effect compared with other models for the CO concentration measurement accuracy of the micro sensor station.

In order to test whether the MLR-BRT-ARIMA combination model proposed in this paper has a good calibration effect on all six types of pollutants in the micro air quality monitor, Root Mean Square Error (RMSE), Mean Absolute Error (MAE) and relative Mean Absolute Percent Error (MAPE) are used to quantitatively compare the calibration effect of each models. Eqn (11)–(13) are expressions of these three evaluation indicators, where *y*_*i*_ represents the reference value and *w*_*i*_ represents the model fitted value.^37,47^

It can be seen from Tables 5–7 that no matter which evaluation index, the index value of the micro sensor station is the largest, indicating that the measurement accuracy of the micro sensor station needs to be improved. All the models mentioned in this paper can be used to calibrate the micro sensor station measurements. The calibration effects of the MLR, MLP, SVR and NARX models need to be improved, while the BRT, MLR-BRT and MLR-BRT-ARIMA models have good calibration effects for various pollutant concentrations, which are basically consistent with the intuitive display results of Taylor diagram. The main reason for the good calibration effect of the BRT, MLR-BRT and MLR-BRT-ARIMA models is due to the high accuracy of the BRT model. In addition, the single BRT model is faster and less resource demanding, so it can also be considered if the data volume is huge or the model accuracy requirement is not very high. No matter what kind of pollutant, the MLR-BRT-ARIMA model proposed in this paper has the best performance in each index. In the RMSE index, the MLR-BRT-ARIMA model of SO_2_ has the best effect on micro sensor station accuracy calibration, the index value is improved from 26.24 to 2.684, and the accuracy is increased by 89.8%. In the MAE index, the MLR-BRT-ARIMA model of PM_10_ has the best effect on micro sensor station accuracy calibration, the index value is improved from 50.151 to 4.033, and the accuracy is increased by 92%. In the MAPE index, the MLR-BRT-ARIMA model of O_3_ has the best effect on micro sensor station accuracy calibration, the index value is improved from 4.322 to 0.198, and the accuracy is increased by 95.4%. On the whole, the MLR-BRT-ARIMA model shows that the lower the accuracy of micro sensor station, the better the model calibration effect.11
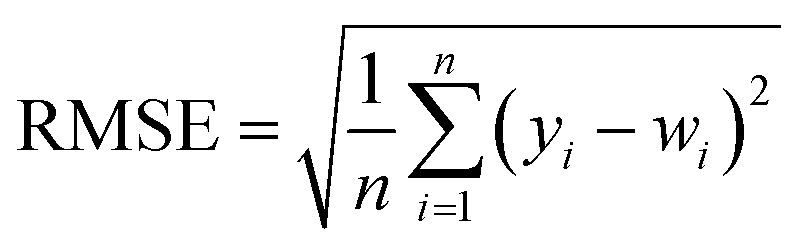
12
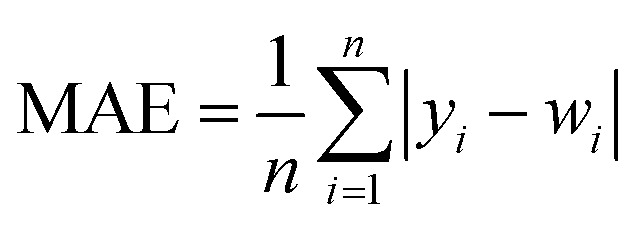
13
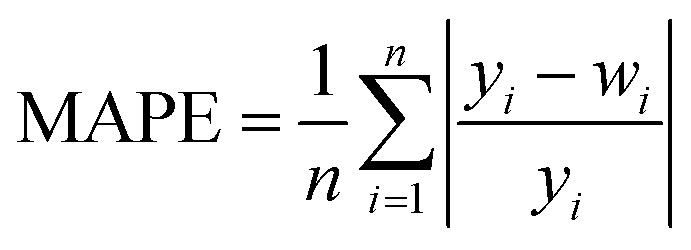


**Table 5:** The RMSE of micro sensor station and various air quality calibration models, in which reference sensor station is used as comparison object

Input variable	Micro sensor station	MLR	MLR-BRT	BRT	MLR-BRT-ARIMA	MLP	SVR	NARX
PM_2.5_	22.436	10.145	3.943	3.946	3.938	10.777	8.649	8.8
PM_10_	66.263	20.036	7.828	8.215	7.729	19.126	11.656	13.911
CO	0.679	0.344	0.101	0.103	0.098	0.304	0.175	0.158
NO_2_	37.183	16.667	4.519	4.673	4.511	13.216	7.725	8.081
SO_2_	26.24	15.31	2.756	2.849	2.684	9.984	4.116	5.104
O_3_	45.673	21.451	6.376	6.564	6.193	18.603	11.304	12.477

**Table 6:** The MAE of micro sensor station and various air quality calibration models, in which reference sensor station is used as comparison object

Input variable	Micro sensor station	MLR	MLR-BRT	BRT	MLR-BRT-ARIMA	MLP	SVR	NARX
PM_2.5_	18.181	7.027	2.361	2.404	2.357	7.763	5.821	6.07
PM_10_	50.151	13.7	4.096	4.338	4.033	13.184	7.08	9.218
CO	0.549	0.263	0.056	0.058	0.055	0.237	0.11	0.1
NO_2_	29.838	12.65	2.506	2.661	2.508	9.991	4.658	4.924
SO_2_	12.867	10.193	1.473	1.529	1.457	7.246	2.116	2.684
O_3_	36.63	16.534	3.685	3.867	3.624	14.396	7.647	7.948

**Table 7:** The MAPE of micro sensor station and various air quality calibration models, in which reference sensor station is used as comparison object

Input variable	Micro sensor station	MLR	MLR-BRT	BRT	MLR-BRT-ARIMA	MLP	SVR	NARX
PM_2.5_	0.447	0.166	0.06	0.061	0.06	0.185	0.133	0.151
PM_10_	0.887	0.222	0.066	0.069	0.065	0.21	0.107	0.147
CO	0.478	0.317	0.058	0.06	0.057	0.283	0.112	0.096
NO_2_	2.129	0.644	0.103	0.112	0.103	0.471	0.17	0.1816
SO_2_	0.685	0.637	0.1	0.104	0.096	0.53	0.131	0.161
O_3_	4.322	1.24	0.203	0.208	0.198	1.002	0.373	0.428

## 5. Conclusions

The development of micro air quality monitors has enabled humans to monitor pollutant concentrations in real time and on a grid basis. However, its measurement accuracy needs to be improved. The MLR-BRT-ARIMA combined model proposed in this paper improves the accuracy of the micro air quality monitor by 82.4–95.4%. This combined model not only gives quantitative relationships between the explained variables and their influencing factors, but also has higher predictive accuracy than the multiple linear regression and boosted regression tree models alone. Using the ARIMA model to correct the residuals can further improve the calibration effect of the model. The establishment of the MLR-BRT-ARIMA combined model is based on 206 days of data from November 2018 to June 2019, including a total of 4135 sets of data, covering four seasons, indicating that the calibration model has strong stability. Moreover, this calibration model has good performance not only in the training set, but also in the test set, indicating that the calibration model has strong generalization ability. However, the influencing factors of air quality are complex, and the establishment of the MLR-BRT-ARIMA combined model does not consider other external factors. Future research can consider introducing more external factors to improve the calibration effect of the model. In addition, different regions have different climatic conditions, and whether this calibration model is suitable for other regions needs to be verified in practice.

## Abbreviations

MLR: Multiple linear regression
BRT: Boosted regression tree
ARIMA: AutoRegressive integrated moving average
RSS: Reference sensor station
MSS: Micro sensor station
MLP: Multi layer perceptron neural network
SVR: Support vector regression machine
NARX: Nonlinear autoRegressive models with eXogenous inputs
RMSE: Root mean square error
MAE: Mean absolute error
MAPE: Mean absolute percent error

## Author contributions

Bing Liu: conceptualization, methodology, validation, formal analysis, writing—original draft preparation, supervision, project administration and funding acquisition. Peijun Jiang: conceptualization, validation, formal analysis and visualization. All authors have read and agreed to the published version of the manuscript.

## Conflicts of interest

There are no conflicts to declare.

## Supplementary Material
